# Discovery of a novel bat lyssavirus in a Long-fingered bat (*Myotis capaccinii*) from Slovenia

**DOI:** 10.1371/journal.pntd.0011420

**Published:** 2023-06-29

**Authors:** Danijela Černe, Peter Hostnik, Ivan Toplak, Primož Presetnik, Jedrt Maurer-Wernig, Urška Kuhar

**Affiliations:** 1 Institute of Microbiology and Parasitology, Virology Unit, Veterinary Faculty, University of Ljubljana, Ljubljana, Slovenia; 2 Centre for Cartography of Fauna and Flora, Ljubljana office, Ljubljana, Slovenia; 3 Administration of the Republic of Slovenia for food safety, veterinary sector, and plant protection, Ljubljana, Slovenia; US Department of Agriculture, UNITED STATES

## Abstract

Lyssaviruses are the causative agents of rabies, a zoonotic, fatal disease that is thought to be ancestral to bats. In the last decade, the detection of bat associated lyssaviruses is increasing also in Europe. Within a retrospective bat associated lyssavirus surveillance study a total of 225 dead bats of 21 bat species were collected in Slovenia between 2012 and 2019 and tested by specific real-time RT-PCR method. The first lyssavirus positive sample in bats in Slovenia was detected using the real-time RT-PCR, the fluorescent antibody test, and next generation sequencing, while the rabies tissue culture inoculation test was unsuccessful due to sample degradation and storage conditions. The nearly complete genome of Divača bat lyssavirus from Slovenia consists of 11,871 nucleotides and reflects the characteristic gene organization known for lyssaviruses, encoding the five viral proteins. Phylogenetic analysis of Divača bat lyssavirus revealed that it belongs to phylogroup I lyssaviruses and is most closely related to Kotalahti bat lyssavirus (KBLV) with 87.20% nucleotide and 99.22% amino acid identity. Together with KBLV, Khujand virus, European bat lyssavirus 2, Bakeloh bat lyssavirus, and Aravan virus, Divača bat lyssavirus was detected in the genus Myotis suggesting its key role in the transmission and maintenance of certain lyssaviruses.

## Author summary

Lyssaviruses are the causative agents for rabies, a zoonotic, fatal disease that is thought to be ancestral to bats. The wide geographical distribution and genetic diversity of lyssaviruses detected in bats across Europe indicated the need for lyssavirus surveillance in the Slovenian bat population. Within a retrospective surveillance study on bat-associated lyssavirus in Slovenia, one positive sample was detected. Whole genome sequencing and phylogenetic analyses revealed that the Divača bat lyssavirus genome, from Slovenia, reflects the characteristic gene organization known for lyssaviruses, its classification in phylogroup I, and its close relation to tentative lyssavirus species KBLV. Together with KBLV, KHUV, EBLV 2, BBLV, and ARAV, Divača bat lyssavirus was detected in the genus Myotis suggesting its key role in the transmission and maintenance of certain lyssaviruses. Worldwide increased laboratory surveillance and pan-lyssavirus molecular tools will support the discovery of novel lyssaviruses which could greatly impact recognizing the risk of lyssaviruses to animal and human health.

## Introduction

Bats belong to the mammalian order *Chiroptera*, with more than 1460 species in 21 bat families [1]. They are widely distributed on all continents except Antarctica and play an important role in seed dispersal, pollination, and in organic fertilization through their guano [2]. Bats are a natural reservoir for viruses that are among the deadliest viruses transmitted from wildlife to humans, including rabies, and SARS coronavirus [3]. As reservoir hosts for emerging viral zoonotic diseases, bats also carry viruses from at least 28 different virus families, most of which are likely host-specific and have limited zoonotic potential [4]. The number of viral zoonoses in bats is comparable to the number of viral zoonoses in other mammal or bird orders since variation in the number of viral zoonoses among animal groups arises as a consequence of their species richness [5].

The first described zoonotic disease associated with bats was rabies [6], a zoonotic fatal disease caused by a member of the genus *Lyssavirus*, within the subfamily *Alpharhabdovirinae*, family *Rhabdoviridae*, order *Mononegavirales* [7]. Lyssaviruses are bullet-shaped enveloped viruses with an approximately 12-kb-long linear single stranded negative-sensed RNA genome encoding nucleoprotein (N), phosphoprotein (P), matrix protein (M), glycoprotein (G), and RNA-dependent polymerase (L) [7,8]. According to the taxonomic classification, the genus *Lyssavirus* currently consists of 17 different virus species. According to the most recent ICTV report [9], lyssavirus names are provided here followed by the traditional abbreviations used to identify their isolates: Aravan virus (ARAV), Australian bat lyssavirus (ABLV), Bokeloh bat lyssavirus (BBLV), West Caucasian bat virus (WCBV), Duvenhage virus (DUVV), Taiwan bat lyssavirus (TWBLV), Gannoruwa bat lyssavirus (GBLV), European bat lyssavirus 1 (EBLV-1), European bat lyssavirus 2 (EBLV-2), Ikoma lyssavirus (IKOV), Irkut virus (IRKV), Khujand virus (KHUV), Lagos bat virus (LBV), Lleida bat lyssavirus (LLEBV), Mokola virus (MOKV), rabies virus (RABV), Shimoni bat virus (SHIBV) [9]. Recently, two potentially novel lyssaviruses, Kotalahti bat lyssavirus (KBLV) and Matlo bat lyssavirus (MBLV) have been described [9–12]. Apart from the two virus species MOKV and IKOV within the lyssavirus genus, all lyssaviruses are associated with bats and bats are thought to be the primary ancestral reservoir hosts [13]. In Europe, spillover lyssavirus infections from insectivorous bats involving EBLV-1, EBLV-2, and WCBV have been described in humans [14–17], cats [18,19], sheep [20], and stone martens [21]. All lyssaviruses are thought to be capable of causing central nervous system infection leading to acute progressive encephalomyelitis [13] and death in unvaccinated humans if not treated appropriately [16].

According to phylogenetic and antigenic characteristics, lyssaviruses are divided into two phylogroups [22]. ABLV, ARAV, BBLV, DUVV, EBLV-1, EBLV-2, GBLV, IRKV, KBLV, KHUV, RABV, TWBLV represent phylogroup I, while LBV, MOKV, and SHIBV represent phylogroup II [22–24]. The classification into phylogroups I and II could not be applied to the most divergent lyssaviruses IKOV, LLEBV, MBLV, and WCBV [25]. The highest nucleotide sequence similarity of the glycoprotein gene was described within the phylogroup II with an average of 71.5%, followed by phylogroup I with 70.3% similarity and the group of lyssaviruses not assigned to phylogroups I and II (IKOV, LLEBV, MBLV, WCBV) with 58.2% similarity [25]. According to Fooks et al. [25], rabies vaccines are not effective against the most divergent lyssaviruses (IKOV, LLEBV, MBLV, WCBV) belonging to the group of lyssaviruses not assigned to phylogroups I and II [25].

In Europe, rabies in bats was first reported in Germany in 1954 [26]. With subsequent molecular analyses of rabies virus genomes in European bats, the distinction between classical RABV and EBLV 1 and 2 was described [27]. In European bats, EBLV-1 was detected in Serotine bat (*Eptesicus serotinus)* and Isabelline serotine bat *(Eptesicus isabellinus)* [27–29]. EBLV-2 was detected in European bat species Daubenton’s bat (*Myotis daubentonii)* and Pond bat (*Myotis dasycneme)* [30], WCBV in Common bent-wing bat (*Miniopterus schreibersii)* [19,31], LLEBV in Common bent-wing bat *(Miniopterus schreibersii)* [32], BBLV in Natterer’s bat (*Myotis nattereri)* [33], and KBLV in Brandt’s bat (*Myotis brandtii)* [34].

In this manuscript, we describe the discovery and genetic characterisation of a previously unknown bat-associated lyssavirus, Divača bat lyssavirus, in an insectivorous bat species from Slovenia as a result of national retrospective surveillance programme. Based on whole genome sequencing and phylogenetic analysis, Divača bat lyssavirus, together with KBLV, may represent a putative new lyssavirus species in Europe.

## Material and methods

### Sampling

Between 2012 and 2019, a total of 225 dead bats of 21 bat species from 59 (out of 212) municipalities in Slovenia were collected by bat biologists or volunteers and stored frozen (S1 Table). The collected dead bats were submitted to the National Veterinary Institute, Institute of Microbiology and Parasitology, Virology unit, for lyssavirus diagnosis. Bat biologists provided information on bat species, location (municipality), and year of collection. Bat species were identified using morphological keyes, described in Dietz and Halversen [35]. Additional molecular characterization of the host species from lyssavirus positive bat sample PP-0868/2014, where NGS reads were mapped to cytochrome b and cytochrome c oxidase subunit I [36], confirmed the original assignment classification to *Myotis capaccinii*.

Brain samples were collected through the foramen occipitale magnum by pipette aspiration. After aspiration of the brain tissue, the cranial cavity was rinsed with RPMI-1640 medium (Thermo Fisher, USA). The collected brain tissue was homogenized in a total volume of 500 μl of RPMI-1640 medium (Thermo Fisher, USA) before long-term storage at < - 60°C.

Permit for capture, disturbance, and temporary taking from the wild and sampling of protected animals was issued by the Agency of the Republic of Slovenia for the Environment No. 35601-35/2010-6.

### Diagnostic methods

For molecular detection of lyssaviruses, brain tissue homogenates were subjected to automated RNA extraction using the KingFisher Flex Purification System (Thermo Fisher, USA) and MagMax Core Nucleic Acid Purification Kit (Thermo Fisher, USA) according to the manufacturer’s instructions. The extracted RNA was tested by real time RT-PCR [37,38] to detect lyssaviruses. Real-time RT-PCR with three specific primers LN34forward1, LN34forward2, and LN34reverse, and two probes LN34probe and LN34probeLago targeting highly conserved sequences within the nucleoprotein gene region [37] was performed.

When the lyssavirus-positive sample PP-0868/2014 was detected by real-time RT-PCR, the bat carcass was resampled by opening the cranial cavity to obtain the brain sample, which was subjected to a fluorescent antibody test (FAT) performed as previously described [39].

RTCIT was performed from lyssavirus positive sample PP-0868/2014 as previously described [39]. Three consecutive serial passages were performed to obtain RTCIT results.

### Whole genome sequencing and phylogenetic analysis

The lyssavirus positive sample PP-0868/2014 was subjected to next generation sequencing (NGS) to determine the complete genome sequence of lyssavirus using the NGS service of Novogene (Cambridge, UK). From extracted RNA to final data, Novogene’s service included sample preparation, quality control, library construction using the RIP-seq library preparation kit (Illumina, USA), library quality control, sequencing on NovaSeq 6000 (Illumina, USA), using the NovaSeq PE150 kit (Illumina, USA), and data quality control. NGS reads were used for *de novo* assembly with SPAdes 3.13.0 [40]. Diamond BLASTx [41] and MEGAN 6.11.7 [42] were used for the taxonomic assignment of the assembled contigs. The nucleotide sequence of the assembled lyssavirus genome, named Divača bat lyssavirus (original name of sample PP-0868/2014), was deposited in the GenBank database with accession number OQ428158 and compared with other genomes of 17 lyssavirus species and 2 putative lyssavirus species and annotated using Geneious 20221.1 (Biomatters, New Zealand). The nucleotide and amino acid alignments were constructed using MAFFT [43]. Phylogenetic analysis was performed using IQ-TREE 1.6.12 [44], with ModelFinder [45] determining the best model according to BIC score and 1000 ultrafast bootstrap replicates [46,47] used to test tree reliability.

## Results

### Virus detection by real-time RT-PCR, FAT, and RTCIT

One sample (original name PP-0868/2014) out of 225 tested samples from 21 different bat species (S1 Table) was found to be lyssavirus positive by the real-time RT-PCR, with a cycle threshold value of 21.82. The sample PP-0868/2014 was from species Long-fingered bat (*Myotis capaccinii)*, which was found dead approximately 700m inside of Škocjan caves in Divača municipality collected in the year 2014. The bat carcass was found in the advance stage of decay and was stored at < - 20°C until brain collection in 2020. The lyssavirus positive brain sample PP-0868/2014 was subjected to further analysis, namely FAT and RTCIT. The FAT slide stained with FITC anti-rabies monoclonal globulin showed positive results with typical lyssavirus staining (S1 Fig). No viral growth was detected on RTCIT for PP-0868/2014 after three consecutive passages.

### Whole genome sequencing and phylogenetic analysis

Viral RNA from lyssavirus positive sample PP-0868/2014 was subjected to NGS to determine the complete genome sequence, named Divača bat lyssavirus. NGS yielded 13,048,710 raw reads that were used for *de novo* assembly. The nearly complete lyssavirus genome sequence with a total length of 11,871 nucleotides (an average sequence depth of 135,333 nucleotides per nucleotide site) was generated, encoding five genes, N, P, M, G, and L in the highly conserved order typical of lyssaviruses. The lengths of the N, P, M, G, and L protein genes were 1356 nt, 894 nt, 609 nt, 1581 nt, and 6384 nt, respectively. Gene lengths and intergenic spacers of Divača bat lyssavirus were found to be similar to all lyssaviruses from phylogroup I.

**Fig 1:**
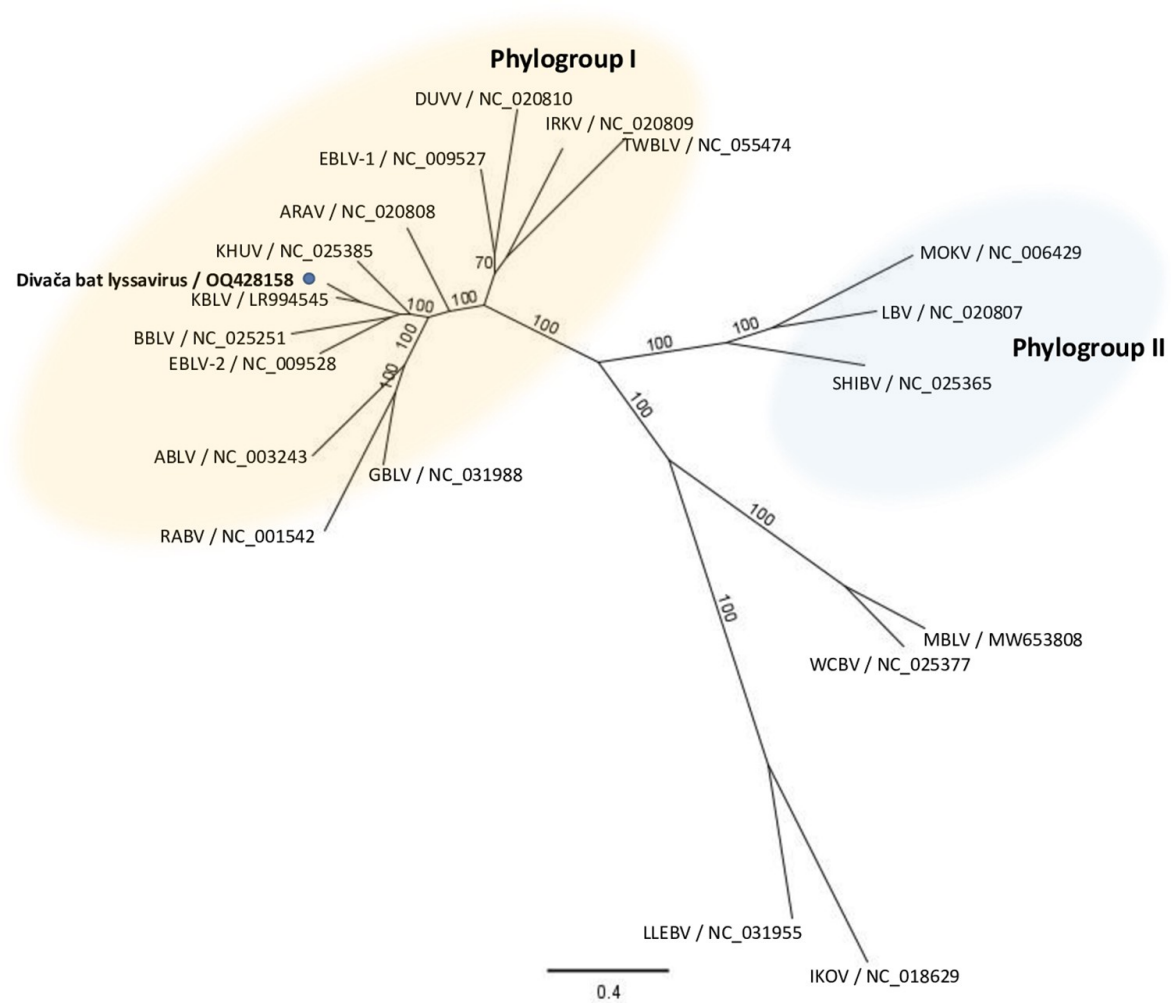
N+P+M+G+L maximum likelihood phylogenetic tree. The maximum likelihood phylogeny was constructed with concatenated N+P+M+G+L coding sequences of representative 17 lyssavirus species, 2 tentative lyssavirus species, and Divača bat lyssavirus (indicated with a blue dot) by IQ-TREE using the GTR+F+I+G4 substitution model with 1000 ultrafast bootstrap replicates. Numbers at the nodes indicate ultrafast bootstrap support and the scale bar indicates the number of substitutions per site.

Phylogenetic analysis of the concatenated N+P+M+G+L coding sequences of the assembled Divača bat lyssavirus, 17 lyssavirus species, and 2 putative lyssavirus species (Fig 1) showed that Divača bat lyssavirus clustered with phylogroup I lyssaviruses (Fig 1) and is most closely related to KBLV with 87.20% nucleotide and 99.22% amino acid identity (Table 1). Similar nucleotide and amino acid identities to KBLV were found for the N gene and also for the P, M, G, and L genes. The percent nucleotide and amino acid identities of the N, P, M, G, L, and concatenated N+P+M+G+L genes between Divača bat lyssavirus and other lyssavirus species from GenBank are shown in Table 1.

**Table 1:** Nt and aa % identity of N, P, M, G, L and concatenated N+P+M+G+L genes of 19 different lyssaviruses to Divača bat lyssavirus.

Lyssavirus species abbrev.	N gene nt/aa % identity to Divača bat lyssavirus	P gene nt/aa % identity to Divača bat lyssavirus	M gene nt/aa % identity to Divača bat lyssavirus	G gene nt/aa % identity to Divača bat lyssavirus	L gene nt/aa % identity to Divača bat lyssavirus	Concatenated N+P+M+G+L genes nt/aa % identity to Divača bat lyssavirus
**ARAV**	79.20/ 97.78	74.61/ 93.27	79.97/ 99.50	74.89/ 95.82	77.79/ 98.35	77.40/ 97.56
**ABLV**	76.72/ 97.78	70.02/ 89.56	77.01/ 98.02	70.91/ 92.00	75.15/ 96.95	74.25/ 95.44
**BBLV**	78.69/ 97.78	77.29/ 94.28	80.13/ 99.01	75.94/ 95.42	79.14/ 98.64	78.47/ 97.67
**WCBV**	71.69/ 95.56	52.81/ 73.91	69.13/ 95.05	55.51/ 76.57	68.34/ 92.99	65.48/ 89.12
**DUVV**	76.18/ 96.67	65.33/ 88.93	77.67/ 98.02	72.19/ 93.73	73.90/ 97.04	73.33/ 95.69
**TWBLV**	74.63/ 96.45	66.78/ 88.26	78.00/ 98.51	66.79/ 91.06	74.17/ 96.94	72.46/ 95.01
**GBLV**	77.97/ 98.00	71.14/ 88.22	76.52/ 99.50	74.38/ 94.49	77.41/ 98.07	76.46/ 96.78
**EBLV-1**	76.99/ 97.12	66.89/ 89.60	75.37/ 97.03	73.71/ 93.51	76.05/ 97.41	74.99/ 96.09
**EBLV-2**	78.61/ 96.45	76.17/ 93.60	79.79/ 98.51	78.29/ 95.99	79.83/ 98.45	79.13/ 97.39
**IKOV**	68.22/ 91.56	49.56/ 73.15	68.14/ 94.06	52.34/ 74.24	65.40/ 90.12	62.47/ 86.50
**IRKV**	77.29/ 96.90	67.78/ 89.93	78.33/ 97.52	70.10/ 94.27	74.94/ 97.27	74.11/ 96.12
**KHUV**	80.31/ 97.34	76.96/ 95.62	78.98/ 99.01	76.60/ 95.06	80.01/ 98.73	79.24/ 97.78
**LBV**	73.39/ 96.22	53.64/ 77.74	72.09/ 96.04	58.89/ 83.91	71.08/ 94.41	67.93/ 91.54
**LLEBV**	70.07/ 92.89	50.44/ 72.73	69.46/ 94.55	52.66/ 74.90	65.52/ 90.55	62.66/ 86.97
**MOKV**	71.54/ 95.78	54.36/ 77.26	71.26/ 96.04	60.42/ 82.95	70.29/ 94.17	67.66/ 91.18
**RABV**	75.76/ 96.89	69.35/ 87.21	71.76/ 96.04	69.71/ 91.60	74.06/ 96.94	73.39/ 95.23
**SHIBV**	72.88/ 95.11	56.35/ 81.33	71.76/ 97.03	60.48/ 83.52	71.19/ 94.22	68.54/ 91.57
**KBLV**	87.09/ 99.78	87.81/ 97.64	89.49/ 100.00	86.40/ 98.10	87.11/ 99.53	87.20/ 99.22
**MBLV**	72.58/ 95.78	52.59/ 71.57	68.31/ 95.05	54.56/ 75.81	68.39/ 92.71	65.41/ 88.79

## Discussion

Several bat lyssavirus surveillance systems have been implemented in Europe and results have shown that passive or retrospective surveillance is better than active surveillance for lyssavirus detection [27,50]. In this study, a retrospective survey was conducted between 2012 and 2019, collecting 225 carcasses from 21 of the 32 bat species living in Slovenia [51]. Lyssavirus was found in one of the two *Myotis capaccinii* carcasses. Based on a single detection of lyssavirus in a particular bat species, we could not determine whether *Myotis capaccinii* is the true reservoir host for the Divača bat lyssavirus. Similar results and conclusions were reported by Nokireki et al. [34] who described the putative new lyssavirus species KBLV in *Myotis brandtii* and also found only one KBLV positive sample. However, the authors of a later study on KBLV [52] suggested that, based on the data that closely related phylogroup I lyssaviruses have also been isolated from bat species of the genus Myotis, *Myotis brandtii* is probably a reservoir host and not infected by spillover infection from another bat species.

This study is the first to report a lyssavirus detected in *Myotis capaccinii*, a bat species in which several viruses from the families Astroviridae (3), Coronaviridae (6), Herpesviridae (2), and Paramyxoviridae (1) have been described [53]. Infection of bats with lyssaviruses occurs worldwide, although different virus species occur in different regions, have co-evolved and are therefore linked to specific bat species [54]. In the Americas, only RABV is associated with bats, whereas in Europe, Africa, Asia, and Australia, the other lyssaviruses predominate without RABV being associated with bats [55]. In Europe, numerous bat species have already been identified as infected with lyssaviruses [56]. However, there is no report of a lyssavirus finding for the bat species *Myotis capaccinii*.

**Fig 2:**
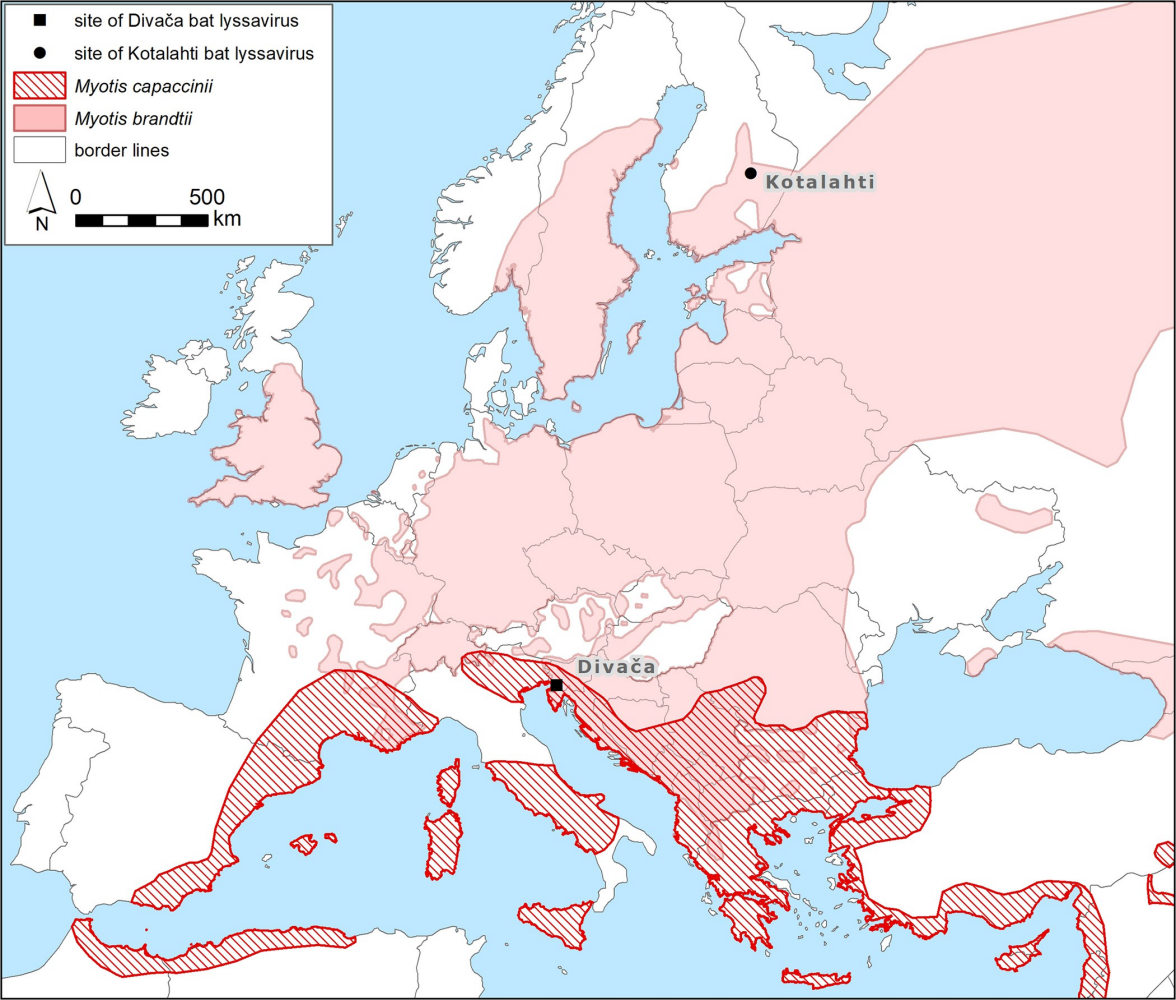
The sites of closely related Divača bat lyssavirus (square) and Kotalahti bat lyssavirus (dot). The geographical distribution of *Myotis capaccinii* and *Myotis brandtii* according to NatureServe and International Union for Conservation of Nature [48,49]. Base layer of the map was made with Natural Earth (https://www.naturalearthdata.com/http//www.naturalearthdata.com/download/10m/cultural/ne_10m_admin_0_sovereignty.zip).

Using NGS, a nearly complete genome sequence of the Divača bat lyssavirus was generated and phylogenetic analysis was performed. According to the ICTV [9], there are several criteria for classifying new species in the genus Lyssavirus. The new species should have a nucleotide identity for the complete N gene of less than 78–80% or a nucleotide identity for the concatenated coding regions of N+P+M+G+L of less than 80%. The Divača bat lyssavirus is most closely related to the putative novel lyssavirus species KBLV with 87.20% and 87.09% nucleotide identity for the concatenated N+P+M+G+L genes and the N gene, respectively, suggesting that Divača bat lyssavirus and KBLV belong to the same putative novel lyssavirus species. Another demarcation criterion of ICTV [9] requires that the new virus should not represent a sister branch to a virus of an established species in the phylogenetic tree of concatenated N+P+M+G+L. Phylogenetic analyses of concatenated N+P+M+G+L revealed that Divača bat lyssavirus represents a sister branch to a putative novel lyssavirus species KBLV, again suggesting that Divača bat lyssavirus and KBLV are members of the same putative novel lyssavirus species. Serological differentiation of a novel lyssavirus species from other lyssavirus species is also one of the species demarcation criteria by ICTV for a novel lyssavirus species, but in our study, Divača bat lyssavirus could not be serologically assessed. The last demarcation criterion of ICTV [9] is that a novel lyssavirus species occupies a distinct ecological niche, as evidenced by host species, pathobiological properties, or geographic range. The Divača bat lyssavirus occupies a specific ecological niche because it originates from a different host than KBLV, which has also been detected in a different geographical location (Fig 2). Taking all four ICTV criteria together to taxonomically classify the novel Divača bat lyssavirus, even though it was found in a new host species almost two thousand kilometres away from the closely related KBLV, we suggest that the Divača bat lyssavirus and KBLV are both members of the same putative novel lyssavirus species.

As already aforementioned, Divača bat lyssavirus is most closely related to KBLV. KBLV was detected in *Myotis brandtii* in Finland in 2017 [34]. In Slovenia, *Myotis brandtii* is very rare, however, it was found in the vicinity of Škocjan caves [57]. The contact between *Myotis capaccinii*, species with circum-Mediterranean distribution [48] almost exclusively roosting in caves, and *Myotis brandtii*, species with wider distribution in Europe [49] primarily roosting in tree holes and cracks, remains unclear as they usually do not share roosts. The geographical distance between Kotalahti in Finland and Divača in Slovenia is quite large, it measures 1886 km (Fig 2). Due to the vast geographical distance and fact that both host species *Myotis brandtii* and *Myotis capaccinii* are mostly regional migrants [58], we could speculate that lyssaviruses similar to KBLV and Divača bat lyssavirus could be present in other European countries between Slovenia and Finland, suggesting the need for surveillance programs and the need to increase public awareness.

Despite efforts to isolate viable virus from the sample PP-0868/2014, we were unsuccessful and could not evaluate the vaccine efficacy of conventional rabies vaccines. However, according to the assignment of Divača bat lyssavirus to phylogroup I and high sequence identity with KBLV we could speculate good cross protection by conventional rabies vaccines.

We believe that the complete genome sequence of Divača bat lyssavirus and its phylogenetic relationships to other lyssaviruses identified in our study may be useful for future studies of the lyssavirus genus. For further studies, the isolation from field samples on cell culture will be an important step toward the possibility of the additional analysis of this strain.

In conclusion, Divača bat lyssavirus is most closely related to KBLV, which has been proposed as a separate species within the genus Lyssavirus. Previously published studies on KBLV indicate that it belongs to phylogroup I of the lyssaviruses against which rabies vaccines provide protection. Continued passive surveillance would be required to obtain infectious lyssaviruses for evaluating the efficacy of vaccines relevant to public health. Because Divača bat lyssavirus has the greatest genetic similarity to KBLV, it could be considered to pose a low risk to public health if preventive measures such as vaccination of bat caretakers and prophylactic treatment after human-bat contact are followed.

## Supporting information

S1 Table Details of sample collection. Lyssavirus positive sample is indicated with blue colour.
(DOCX)

S1 Fig FAT result of Lyssavirus positive sample (PP-0868/2014). Apple green fluorescence is present in neurons (magnification 20×0.40).
(DOCX)
