# Supplementary material for: Discovery of a novel bat lyssavirus in a Long-fingered bat (Myotis capaccinii) from Slovenia
Source: PLoS Negl Trop Dis. 2023 Jun 29;17(6):e0011420. doi: 10.1371/journal.pntd.0011420 (PMC10309629; doi:10.1371/journal.pntd.0011420)
Supplement: S1 Table — Lyssavirus positive sample is indicated with blue colour. (DOCX) [file pntd.0011420.s001.docx]

**Supporting information**

**S1 Table.** **Details of sample collection.** Lyssavirus positive sample is indicated with blue colour.

| Sample name | bat species | municipality | year of sampling |
| --- | --- | --- | --- |
| PP-0853 | *Pipistrellus pygmaeus* | Ljubljana | 2012 |
| PP-0795 | *Pipistrellus kuhlii* | Celje | 2013 |
| PP-0796 | *Nyctalus noctula* | Ljubljana | 2013 |
| PP-0797 | *Pipistrellus kuhlii* | Trbovlje | 2013 |
| PP-0798 | *Pipistrellus nathusii* | Ljubljana | 2013 |
| PP-0799 | *Nyctalus noctula* | Šempeter pri Novi Gorici | 2013 |
| PP-0800 | *Myotis mystacinus* s.l. | Ljubljana | 2013 |
| PP-0801 | *Barbastella barbastellus* | Ljubljana | 2013 |
| PP-0802 | *Pipistrellus kuhlii* | Ljubljana | 2013 |
| PP-0803 | *Pipistrellus nathusii* | Naklo | 2013 |
| PP-0805 | *Pipistrellus nathusii* | Ljubljana | 2013 |
| PP-0817 | *Pipistrellus nathusii* | Maribor | 2013 |
| PP-0818 | *Rhinolophus hipposideros* | Naklo | 2013 |
| PP-0820 | *Pipistrellus nathusii* | Ljubljana | 2013 |
| PP-0823 | *Pipistrellus kuhlii* | Maribor | 2013 |
| PP-0825 | *Pipistrellus nathusii* | Cerknica | 2013 |
| PP-0826 | *Pipistrellus kuhlii* | Ljubljana | 2013 |
| PP-0828 | *Myotis mystacinus* s.l. | Bled | 2013 |
| PP-0837 | *Pipistrellus nathusii* | Ljubljana | 2013 |
| PP-0840 | *Hypsugo savii* | Maribor | 2013 |
| PP-0842 | *Pipistrellus pygmaeus* | Ljubljana | 2013 |
| PP-0843 | *Pipistrellus nathusii* | Ljubljana | 2013 |
| PP-0844 | *Pipistrellus pygmaeus* | Domžale | 2013 |
| PP-0849 | *Pipistrellus pygmaeus* | Ljubljana | 2013 |
| PP-0850 | *Pipistrellus pygmaeus* | Ljubljana | 2013 |
| PP-0851 | *Pipistrellus kuhlii* | Vrhnika | 2013 |
| PP-0860 | *Vespertilio murinus* | Radovljica | 2013 |
| PP-0865 | *Vespertilio murinus* | Maribor | 2013 |
| PP-0874 | *Pipistrellus nathusii* | Vrhnika | 2013 |
| PP-0895 | *Pipistrellus pipistrellus* | Škofja Loka | 2013 |
| PP-0927 | *Hypsugo savii* | Ljubljana | 2013 |
| PP-0806 | *Myotis emarginatus* | Ljubljana | 2014 |
| PP-0807 | *Pipistrellus pygmaeus* | Naklo | 2014 |
| PP-0808 | *Pipistrellus pygmaeus* | Bled | 2014 |
| PP-0809 | *Nyctalus noctula* | Ljubljana | 2014 |
| PP-0810 | *Nyctalus leisleri* | Bovec | 2014 |
| PP-0811 | *Hypsugo savii* | Ljubljana | 2014 |
| PP-0812 | *Vespertilio murinus* | Tržič | 2014 |
| PP-0813 | *Pipistrellus nathusii* | Ljubljana | 2014 |
| PP-0814 | *Rhinolophus hipposideros* | Podčetrtek | 2014 |
| PP-0815 | *Rhinolophus euryale* | Krško | 2014 |
| PP-0830 | *Pipistrellus nathusii* | Ljubljana | 2014 |
| PP-0831 | *Myotis mystacinus* s.l. | Vodice | 2014 |
| PP-0832 | *Pipistrellus kuhlii* | Piran | 2014 |
| PP-0836 | *Pipistrellus nathusii* | Ljubljana | 2014 |
| PP-0838 | *Eptesicus serotinus* | Ljubljana | 2014 |
| PP-0839 | *Myotis daubentonii* | Novo Mesto | 2014 |
| PP-0846 | *Pipistrellus kuhlii* | Ljubljana | 2014 |
| PP-0847 | *Pipistrellus nathusii* | Škofljica | 2014 |
| PP-0848 | *Pipistrellus kuhlii* | Ljubljana | 2014 |
| PP-0863 | *Barbastella barbastellus* | Trebnje | 2014 |
| PP-0864 | *Pipistrellus kuhlii* | Izola | 2014 |
| PP-0866 | *Eptesicus serotinus* | Brezovica pri Ljubljani | 2014 |
| PP-0867 | *Pipistrellus kuhlii* | Maribor | 2014 |
| PP-0868 | *Myotis capaccinii* | Divača | 2014 |
| PP-0870 | *Plecotus macrobullaris* | Črnomelj | 2014 |
| PP-0876 | *Nyctalus noctula* | Ljubljana | 2014 |
| PP-0877 | *Nyctalus noctula* | Ljubljana | 2014 |
| PP-0878 | *Pipistrellus pygmaeus* | Ljubljana | 2014 |
| PP-0879 | *Myotis myotis* | Ljubljana | 2014 |
| PP-0880 | *Plecotus auritus* | Maribor | 2014 |
| PP-0894 | *Myotis mystacinus* s.l. | Škofja Loka | 2014 |
| PP-0896 | *Pipistrellus nathusii* | Mojstrana | 2014 |
| PP-0897 | *Hypsugo savii* | Jesenice | 2014 |
| PP-0924 | *Pipistrellus sp.* | Naklo | 2014 |
| PP-0925 | *Myotis mystacinus* s.l. | Radovljica | 2014 |
| PP-0926 | *Myotis mystacinus* s.l. | Škofja Loka | 2014 |
| PP-0929 | *Myotis mystacinus* s.l. | Domžale | 2014 |
| PP-0942 | *Myotis myotis* | Dravograd | 2014 |
| PP-1008 | *Nyctalus leisleri* | Nova Gorica | 2014 |
| PP-0881 | *Pipistrellus nathusii* | Ljubljana | 2015 |
| PP-0882 | *Myotis emarginatus* | Vinica | 2015 |
| PP-0883 | *Barbastella barbastellus* | Grad | 2015 |
| PP-0890 | *Pipistrellus kuhlii* | Novo mesto | 2015 |
| PP-0898 | *Nyctalus noctula* | Ljubljana | 2015 |
| PP-0899 | *Nyctalus noctula* | Ljubljana | 2015 |
| PP-0900 | *Nyctalus noctula* | Ljubljana | 2015 |
| PP-0901 | *Nyctalus noctula* | Ljubljana | 2015 |
| PP-0902 | *Nyctalus noctula* | Ljubljana | 2015 |
| PP-0903 | *Nyctalus noctula* | Ljubljana | 2015 |
| PP-0904 | *Nyctalus noctula* | Ljubljana | 2015 |
| PP-0905 | *Nyctalus noctula* | Ljubljana | 2015 |
| PP-0906 | *Nyctalus noctula* | Ljubljana | 2015 |
| PP-0907 | *Nyctalus noctula* | Ljubljana | 2015 |
| PP-0908 | *Nyctalus noctula* | Ljubljana | 2015 |
| PP-0909 | *Nyctalus noctula* | Ljubljana | 2015 |
| PP-0910 | *Nyctalus noctula* | Ljubljana | 2015 |
| PP-0911 | *Nyctalus noctula* | Ljubljana | 2015 |
| PP-0912 | *Nyctalus noctula* | Ljubljana | 2015 |
| PP-0913 | *Nyctalus noctula* | Ljubljana | 2015 |
| PP-0914 | *Nyctalus noctula* | Ljubljana | 2015 |
| PP-0915 | *Nyctalus noctula* | Ljubljana | 2015 |
| PP-0916 | *Nyctalus noctula* | Ljubljana | 2015 |
| PP-0917 | *Nyctalus noctula* | Ljubljana | 2015 |
| PP-0918 | *Nyctalus noctula* | Ljubljana | 2015 |
| PP-0919 | *Nyctalus noctula* | Ljubljana | 2015 |
| PP-0920 | *Nyctalus noctula* | Ljubljana | 2015 |
| PP-0921 | *Nyctalus noctula* | Ljubljana | 2015 |
| PP-0922 | *Nyctalus noctula* | Ljubljana | 2015 |
| PP-0923 | *Nyctalus noctula* | Ljubljana | 2015 |
| PP-0930 | *Nyctalus noctula* | Ljubljana | 2015 |
| PP-0931 | *Nyctalus noctula* | Ljubljana | 2015 |
| PP-0932 | *Nyctalus noctula* | Ljubljana | 2015 |
| PP-0936a | *Pipistrellus pipistrellus* | Kamnik | 2015 |
| PP-0936b | *Pipistrellus pipistrellus* | Kamnik | 2015 |
| PP-0936c | *Pipistrellus pipistrellus* | Kamnik | 2015 |
| PP-0936d | *Pipistrellus pipistrellus* | Kamnik | 2015 |
| PP-0941 | *Myotis myotis* | Divača | 2015 |
| PP-0943 | *Eptesicus serotinus* | Vipava | 2015 |
| PP-0946 | *Rhinolophus hipposideros* | Cerknica | 2015 |
| PP-0947 | *Pipistrellus kuhlii* | Ljubljana | 2015 |
| PP-0948 | *Pipistrellus nathusii* | Ljubljana | 2015 |
| PP-0950 | *Pipistrellus kuhlii* | Lukovica | 2015 |
| PP-0951 | *Pipistrellus nathusii* | Ljubljana | 2015 |
| PP-0954 | *Plecotus auritus* | Medvode | 2015 |
| PP-0955 | *Pipistrellus kuhlii* | Celje | 2015 |
| PP-0956 | *Myotis mystacinus* s.l. | Ljubljana | 2015 |
| PP-0958 | *Hypsugo savii* | Ljubljana | 2015 |
| PP-0959 | *Pipistrellus pygmaeus* | Ljubljana | 2015 |
| PP-0960 | *Pipistrellus kuhlii* | Ljubljana | 2015 |
| PP-0961 | *Pipistrellus nathusii* | Ljubljana | 2015 |
| PP-0962 | *Pipistrellus pipistrellus* | Kamnik | 2015 |
| PP-0963 | *Pipistrellus nathusii* | Ljubljana | 2015 |
| PP-0964 | *Rhinolophus hipposideros* | Novo mesto | 2015 |
| PP-0965 | *Pipistrellus kuhlii* | Ljubljana | 2015 |
| PP-0966 | *Pipistrellus nathusii* | Ljubljana | 2015 |
| PP-0967 | *Pipistrellus nathusii* | Ljubljana | 2015 |
| PP-0968 | *Myotis mystacinus* s.l. | Tržič | 2015 |
| PP-0969 | *Pipistrellus sp.* | Kranj | 2015 |
| PP-0970 | *Pipistrellus nathusii* | Radovljica | 2015 |
| PP-0971 | *Myotis mystacinus* s.l. | Kranj | 2015 |
| PP-0972 | *Pipistrellus nathusii* | Kranj | 2015 |
| PP-0973 | *Pipistrellus pipistrellus* | Tržič | 2015 |
| PP-0976 | *Pipistrellus nathusii* | Maribor | 2015 |
| PP-0977 | *Myotis bechsteinii* | Ljutomer | 2015 |
| PP-0978 | *Myotis mystacinus* s.l. | Domžale | 2015 |
| PP-0979 | *Pipistrellus kuhlii* | Ljubljana | 2015 |
| PP-0982 | *Pipistrellus pygmaeus* | Ljubljana | 2015 |
| PP-0984 | *Pipistrellus kuhlii* | Krško | 2015 |
| PP-0985 | *Vespertilio murinus* | Ljubljana | 2015 |
| PP-0987 | *Pipistrellus kuhlii* | Ljubljana | 2015 |
| PP-0988 | *Pipistrellus kuhlii* | Sevnica | 2015 |
| PP-0998 | *Nyctalus noctula* | Ljubljana | 2016 |
| PP-0983 | *Pipistrellus sp.* | Ljubljana | 2016 |
| PP-0989a | *Rhinolophus hipposideros* | Kamnik | 2016 |
| PP-0990 | *Pipistrellus kuhlii* | Ljutomer | 2016 |
| PP-0991 | *Myotis emarginatus* | Borovnica | 2016 |
| PP-0995 | *Vespertilio murinus* | Dravograd | 2016 |
| PP-0966 | *Nyctalus noctula* | Ljubljana | 2016 |
| PP-1000 | *Rhinolophus hipposideros* | Idrija | 2016 |
| PP-1002 | *Myotis myotis* | Vrhnika | 2016 |
| PP-1004 | *Pipistrellus nathusii* | Ljubljana | 2016 |
| PP-1005 | *Pipistrellus nathusii* | Ljubljana | 2016 |
| PP-1006 | *Hypsugo savii* | Ljubljana | 2016 |
| PP-1007 | *Vespertilio murinus* | Ljubljana | 2016 |
| PP-1009 | *Nyctalus noctula* | Vrhnika | 2016 |
| PP-1010 | *Myotis myotis* | Ljutomer | 2016 |
| PP-1011 | *Pipistrellus nathusii* | Ljubljana | 2016 |
| PP-1012 | *Pipistrellus pygmaeus* | Ljubljan | 2016 |
| PP-1013 | *Hypsugo savii* | Ljubljan | 2016 |
| PP-1014 | *Eptesicus serotinus* | Logatec | 2016 |
| PP-1015 | *Plecotus macrobullaris* | Ljubljana | 2016 |
| PP-1016 | *Myotis mystacinus* s.l. | Koper | 2016 |
| PP-1017 | *Vespertilio murinus* | Mozirje | 2016 |
| PP-1018 | *Pipistrellus pygmaeus* | Ljubljana | 2016 |
| PP-1019 | *Pipistrellus nathusii* | Ljubljana | 2016 |
| PP-1020 | *Hypsugo savii* | Ljubljana | 2016 |
| PP-1021 | *Hypsugo savii* | Ljubljana | 2016 |
| PP-1022 | *Pipistrellus sp.* | Naklo | 2016 |
| PP-1023 | *Pipistrellus nathusii* | Ljubljana | 2016 |
| PP-1025 | *Pipistrellus sp.* | Naklo | 2016 |
| PP-1026 | *Pipistrellus pipistrellus* | Kranj | 2016 |
| PP-1027 | *Pipistrellus nathusii* | Kranj | 2016 |
| PP-1028 | *Vespertilio murinus* | Kamnik | 2016 |
| PP-1035 | *Myotis myotis* | Vrhnika | 2016 |
| PP-1042 | *Pipistrellus pipistrellus* | Ljubljana | 2016 |
| PP-1078 | *Vespertilio murinus* | Škofja Loka | 2016 |
| PP-0997 | *Rhinolophus hipposideros* | Logatec | 2017 |
| PP-1029 | *Rhinolophus hipposideros* | Grosuplje | 2017 |
| PP-1030 | *Plecotus macrobullaris* | Kranj | 2017 |
| PP-1032 | *Myotis myotis* | Šmarje pri Jelšah | 2017 |
| PP-1033 | *Pipistrellus kuhlii* | Ljubljana | 2017 |
| PP-1034 | *Pipistrellus kuhlii* | Maribor | 2017 |
| PP-1036 | *Pipistrellus kuhlii* | Vransko | 2017 |
| PP-1037 | *Rhinolophus ferrumequinum* | Metlika | 2017 |
| PP-1044 | *Vespertilio murinus* | Ljubljana | 2017 |
| PP-1045 | *Pipistrellus nathusii* | Ljubljana | 2017 |
| PP-1046 | *Myotis capaccinii* | Divača | 2017 |
| PP-1049 | *Pipistrellus kuhlii* | Rače-Fram | 2017 |
| PP-1050 | *Vespertilio murinus* | Zgornja Polskava | 2017 |
| PP-1063 | *Pipistrellus nathusii* | Ljubljana | 2017 |
| PP-1094 | *Pipistrellus kuhlii* | Gornji Petrovci | 2017 |
| PP-1051 | *Rhinolophus hipposideros* | Mislinaja | 2018 |
| PP-1052 | *Myotis mystacinus* s.l. | Bohinj | 2018 |
| PP-1053 | *Pipistrellus kuhlii* | Kranj | 2018 |
| PP-1057 | *Pipistrellus pipistrellus* | Postojna | 2018 |
| PP-1058 | *Pipistrellus pipistrellus* | Postojna | 2018 |
| PP-1060 | *Vespertilio murinus* | Bloke | 2018 |
| PP-1065 | *Pipistrellus pygmaeus* | Ljubljana | 2018 |
| PP-1066 | *Nyctalus noctula* | Postojna | 2018 |
| PP-1069 | *Hypsugo savii* | Piran | 2018 |
| PP-1071 | *Pipistrellus kuhlii* | Ljubljana | 2018 |
| PP-1072 | *Pipistrellus nathusii* | ljubljana | 2018 |
| PP-1073 | *Pipistrellus kuhlii* | Litija | 2018 |
| PP-1075 | *Pipistrellus nathusii* | Maribor | 2018 |
| PP-1076 | *Rhinolophus hipposideros* | Šoštanj | 2018 |
| PP-1077 | *Rhinolophus hipposideros* | Šoštanj | 2018 |
| PP-1080 | *Pipistrellus pygmaeus* | Ljubljana | 2018 |
| PP-1081 | *Pipistrellus pygmaeus* | Ljubljana | 2018 |
| PP-1082 | *Pipistrellus pipistrellus* | Kamnik | 2018 |
| PP-1083 | *Plecotus macrobullaris* | Ig | 2018 |
| PP-1084 | *Plecotus macrobullaris* | Sežana | 2018 |
| PP-1085 | *Myotis mystacinus* s.l. | Središče ob Dravi | 2018 |
| PP-1093 | *Pipistrellus kuhlii* | Ljubljana | 2019 |
| PP-1098 | *Pipistrellus nathusii* | Ljubljana | 2019 |
| PP-1099 | *Pipistrellus nathusii* | Ljubljana | 2019 |
| PP-1100 | *Rhinolophus hipposideros* | Ivančna Gorica | 2019 |
| PP-1101 | *Rhinolophus hipposideros* | Grosuplje | 2019 |
| PP-1103 | *Eptesicus serotinus* | Šalovci | 2019 |
| PP-1105 | *Pipistrellus kuhlii* | Domžale | 2019 |
| PP-1106 | *Pipistrellus nathusii* | Ljubljana | 2019 |
| PP-1107 | *Rhinolophus hipposideros* | Trebnje | 2019 |
| PP-1108 | *Myotis mystacinus* s.l. | Grosuplje | 2019 |
| PP-1109 | *Hypsugo savii* | Divača | 2019 |
| PP-1111 | *Pipistrellus kuhlii* | Lukovica | 2019 |
