# Supplementary material for: Discovery of a novel bat lyssavirus in a Long-fingered bat (Myotis capaccinii) from Slovenia
Source: PLoS Negl Trop Dis. 2023 Jun 29;17(6):e0011420. doi: 10.1371/journal.pntd.0011420 (PMC10309629; doi:10.1371/journal.pntd.0011420)

**S1 Fig.** **FAT result of Lyssavirus positive sample (PP-0868/2014).** Apple green fluorescence is present in neurons (magnification 20×0.40).


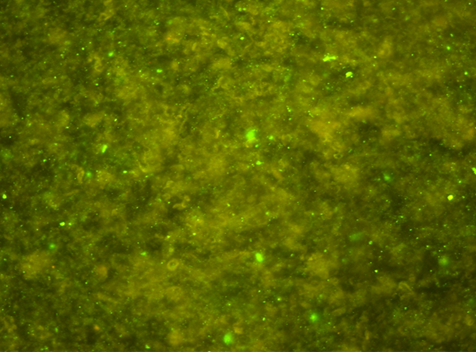

Supplement: S1 Fig — Apple green fluorescence is present in neurons (magnification 20×0.40). (DOCX) [file pntd.0011420.s002.docx]
